# Natural Weathering Effects on the Mechanical, Rheological, and Morphological Properties of Magnetorheological Elastomer (MRE) in Tropical Climate

**DOI:** 10.3390/ijms23179929

**Published:** 2022-09-01

**Authors:** Mohd Aidy Faizal Johari, Saiful Amri Mazlan, Ubaidillah Ubaidillah, Nur Azmah Nordin, Muntaz Hana Ahmad Khairi, Siti Aishah Abdul Aziz, Michal Sedlacik, Siti Asma’ Nikmat Leong

**Affiliations:** 1 Engineering Materials & Structures (eMast) Ikohza, Malaysia-Japan International Institute of Technology (MJIIT), Universiti Teknologi Malaysia, Kuala Lumpur 54100, Malaysia; 2 Mechanical Engineering Department, Faculty of Engineering, Universitas Sebelas Maret, Surakarta 57126, Indonesia; 3 Faculty of Applied Sciences, Universiti Teknologi MARA Pahang, Bandar Tun Abdul Razak Jengka 26400, Malaysia; 4 Centre of Polymer Systems, University Institute, Tomas Bata University in Zlín, Trida T. Bati 5678, 760 01 Zlín, Czech Republic

**Keywords:** durability, environmental stress, magnetorheological elastomer, natural weathering, ozone microcracking, photodegradation, photo-oxidation, ultraviolet

## Abstract

Magnetorheological elastomer (MRE) materials have the potential to be used in a wide range of applications that require long-term service in hostile environments. These widespread applications will result in the emergence of MRE-specific durability issues, where durability refers to performance under in-service environmental conditions. In response, the outdoor tropical climatic environment, combined with the effects of weathering, will be the primary focus of this paper, specifically the photodegradation of the MRE. In this study, MRE made of silicone rubber (SR) and 70 wt% micron-sized carbonyl iron particles (CIP) were prepared and subjected to mechanical and rheological testing to evaluate the effects under natural weathering. Magnetorheological elastomer samples were exposed to the natural weathering conditions of a tropical climate in Kuala Lumpur, Malaysia, for 30 days. To obtain a comprehensive view of MRE degradation during natural weathering, mechanical testing, rheology, and morphological evaluation were all performed. The mechanical and rheological properties test results revealed that after 30 days of exposure and known meteorological parameters, Young’s modulus and storage modulus increased, while elongation at break decreased. The degradation processes of MRE during weathering, which are responsible for their undesirable change, were given special attention. With the help of morphological evidence, the relationship between these phenomena and the viscoelastic properties of MRE was comprehensively defined and discussed.

## 1. Introduction

The advancement of materials technology heralds the emergence of more research and scientific study to accommodate the rapid development of technology and smarter systems. The progression of smart materials research and development around the world has shown that these materials have a high potential for application and contribution to the new evolution of engineering materials. Correspondingly, smarter materials are increasingly being used in new engineering applications; however, this encouraging trend necessitates additional research at the very least to catch up with the conventional materials to date [1]. Magnetorheological elastomer (MRE), for example, is gaining popularity due to numerous advantages in elastic polymer and conventional rubber applications, therewithal its intelligent behavior and changing properties in response to magnetic stimulation [2,3,4,5,6]. The perspective for MRE to be employed in various service environments necessitates an understanding of MRE for specific limitations, and it must undoubtedly be adaptable and durable to meet these challenges. Automotive components, building and bridge design, adaptive vibration isolators, and MRE-based valve actuators are all possible applications. These primarily replace the function of existing applications that use traditional rubber and elastomers, particularly those exposed to weathering.

Magnetorheological elastomer is known to be highly susceptible to temperature [7,8], humidity [9], heat [10,11], chemical attack [12], and moisture [13], all of which degrade performance [14]. Furthermore, while MRE properties can be degraded incrementally during fabrication, operation, and storage, environmental exposure can cause the most significant degradation. The outdoors [15] is one of the most demanding service environments, with weather conditions having a strong correlation. Weathering [16,17,18,19] accelerates the undesirable change caused by outdoor exposure, and the climate atmosphere influences the weathering condition over relatively long periods. As the MRE industry has evolved, significant effort has been expended to understand the degradation of MRE during weathering in various climates, with the focus on tropical climates.

Weathering’s adverse consequences on MRE, in general, have been attributed to a complex set of processes involving environmental stress (photophysical and photochemical). The most recent study on MRE performance under weathering conditions by Wibowo et al. [17] found that MRE microstructurally degraded and rheological properties were significantly reduced up to 80.5%. The UV and water degradation processes that occurred as a result of the breaking of the chain of particle bonds arranged in the MRE sample were also considered. However, it was carried out under accelerated weathering, which was the simulation of natural conditions using extreme weathering conditions or climate fluctuations to accelerate the process [18,19,20]. As a result, the data might be insufficient to comprehend and represent the early-stage behavior of the sample under weathering performance. Wherefore, research into this critical initial period of MRE due to weathering conditions is critical to comprehend the exclusive degradation process towards natural weathering, which, to the best of the author’s knowledge, is in its infancy.

Natural weathering investigations [21,22,23] were carried out on similar materials with elastomeric properties, and the degradation process was thoroughly discussed and analogous to the MRE hypothesis in related studies. Ismail et al. [21,22] discovered that after 3 and 6 months of exposure, the mechanical properties of the sample decreased in elongation at break (EB) while increasing in Young’s modulus. The studies’ microstructure observations revealed that the increase in crystallinity [23,24] was caused by the photo-oxidation [25] of segmented molecules of entangled molecules in the amorphous phase, as well as the ensuing deterioration of mechanical properties. The combined action of light radiation and oxygen in photo-oxidation degradation caused chain scissoring [24] in the amorphous molecular structure, resulting in increased brittleness. Furthermore, other degradations that ought to be considered include UV light degradation [23] and ozone [20]. Studies [26,27,28] on the effects of UV and ozone have significantly influenced mechanical performance and degraded materials microstructurally, primarily due to the presence of oxygen and light radiation in the surroundings.

There is currently no accepted climate classification for the degradation of MRE. Investigation into weathering under specific climate conditions will lead to significant discoveries of MRE characteristics toward natural weathering and its resistance to degradation processes such as photodegradation, thermal degradation, biodegradation, and a few related factors influencing degradation such as oxygen, humidity, light, ozone, and UV radiation. Reciprocally to this challenge, this present study focuses on a variety of factors, particularly environmental stress, which includes photodegradation from sunlight, rainfall, and ambient humidity in a tropical climate. A round-robin test in a similar aspect could be critical in determining the viability of MRE for use in industries with distinct environmental exposure challenges. Furthermore, understanding the characteristics and performance of MREs in relation to the environment can contribute to the new development of better and more reliable MREs that are more resistant, sustainable, and durable to the environment. As a result, the goals of this study are to investigate the effect of natural weathering on the durability properties of MRE by thoroughly reporting on the mechanical, rheological, and morphological behavior toward tropical climate weathering.

## 2. Results and Discussion

### 2.1. Natural Weathering’s Influence on Surface Morphology

**Figure 1:**
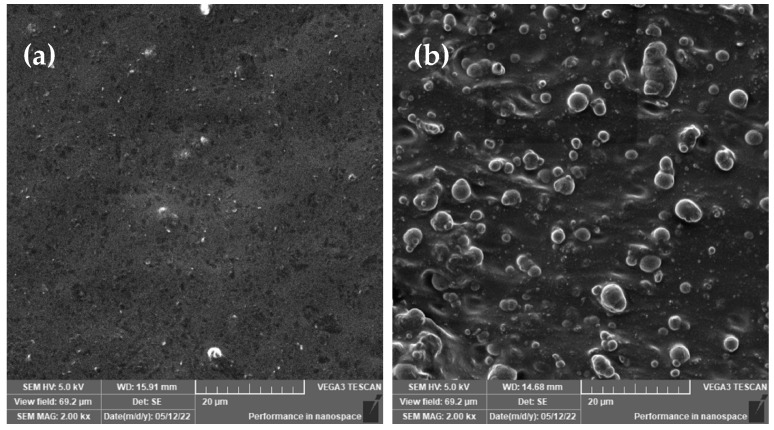
SEM micrographs of (**a**) MRE sample surface morphology, and (**b**) MRE sample cross-section.

Fabricated samples were morphologically analyzed for any defects or deficiencies that occurred during the fabrication process. These included bare silicone rubber (SR) and MRE samples. Figure 1 depicts a surface and cross-section micrograph of MRE samples. The SR sample was discovered to have uniform leveled smooth surfaces similar to the cross-sectional surfaces. Further LV-SEM analysis of the MRE surface reveals a smooth with some sign of CIP lump surface, as shown in Figure 1a, and CIPs embedded with some agglomerations in the matrix of cross-sectioned MRE, as shown in Figure 1b. Carbonyl iron particles were uniformly distributed and clearly showed the isotropic condition of the sample.

**Figure 2:**
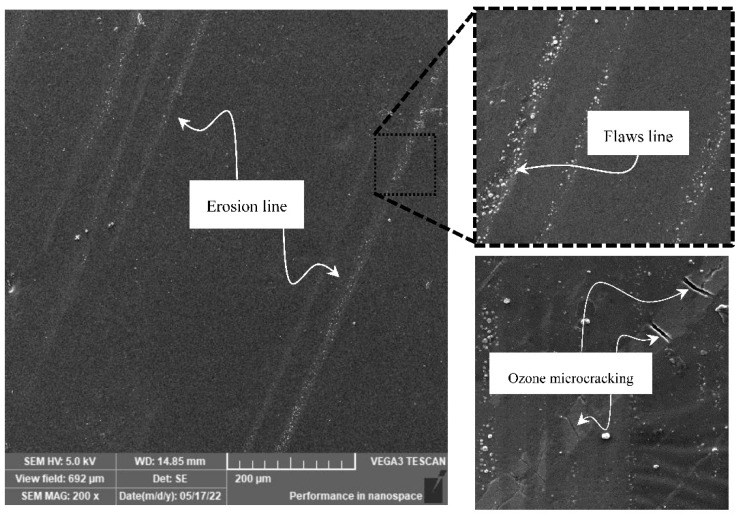
SEM micrographs of MRE samples after 30 days of weathering durability.

However, after 30 days of exposure, the MRE sample developed a surface defect as in Figure 2. Environmental stress was suspected to be influencing the flaws, which could be precipitated by a variety of factors such as photo-oxidation and sudden rainfall, which promoted the effect of thermal stress. The defect, was unique to the MRE sample because CIP was embedded in its elastomeric matrix. On the surface, an erosion line was observed, and this phenomenon was scattered with several parallel lines. The micro-sized eroded line showed a sign of a CIP facet on the surface, which was thought to be the beginning or initiation of ozone cracking. The onset of microcracking in the erosion line was assumed to have progressed along with environmental stress, eventually establishing ozone cracking [26]. The degradation of the MRE surface due to natural weathering was important to understand as it concomitantly manifests the expected service life of the MRE. Further investigation using FESEM led to the conclusion that the degradation could be classified as photodegradation, which was the process of material alteration caused by the interaction of sunlight and air. Degradation typically began at the surface and progresses into the bulk.

### 2.2. Fourier-Transform Infrared Spectral Analysis

**Figure 3:**
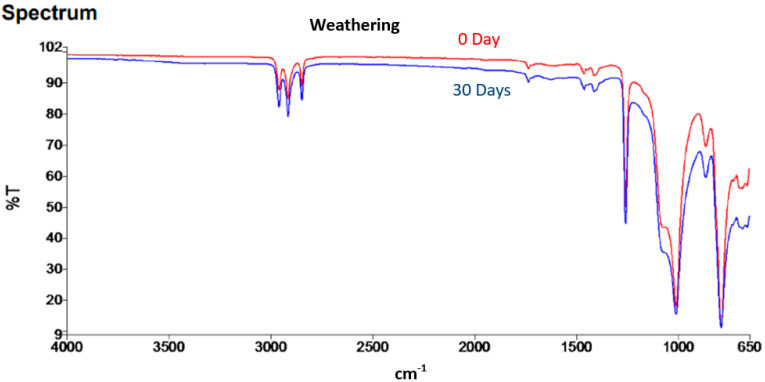
FTIR Spectral of MRE under natural weathering.

Figure 3 depicts the results of FTIR testing on test samples, which produced data in the form of a graph of wavelength (cm^−^^1^) vs. % transmission. The graph shows the presence of transmission as a peak that interpreted the bonding’s characteristic. The bonds formed in test specimens before and after the natural weathering durability procedure were depicted in this interpretation. In general, the sample showed no discernible changes, and the intensity of bond formation appeared to be the same in both conditions. However, in the FTIR graph, the peak high changes, indicating that the peak intensity decreased after 30 days. This indicated a decrease in the amount of the functional group associated with the molecular bond (per unit volume).

### 2.3. Natural Weathering’s Influence on Mechanical Properties

**Figure 4:**
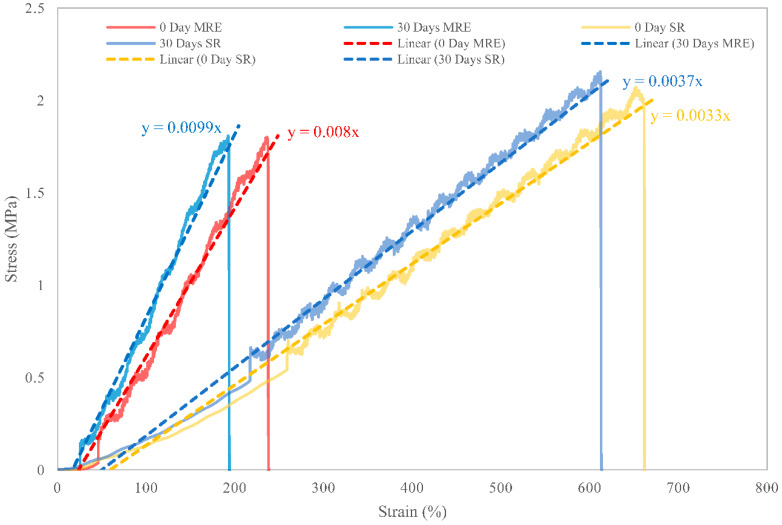
Stress–strain curves of SR and MRE samples, before and after 30 days of natural weathering exposure, and linear regression on the increment slope.

The mechanical properties of MRE were evaluated using a uniaxial tensile test for an original state and after 30 days of natural weathering. The test was also carried out on a bare SR sample, which was similar to the materials used in the MRE matrix system, to understand the effect of weathering on the matrix itself. Prior to the weather durability test, amorphous molecular structures of the matrix were formed, with initial cross-linkage positions generated after curing with the curing agent. The cross-linking produced by the process has a long and entangled amorphous structure that allowed it to be stretched to its maximum elongation [29]. As a result of its high elasticity, the SR sample exhibited the greatest elongation in its original state as shown in Figure 4. The elasticity and deformation limits of MRE were determined by cross-linkages formed during the curing process. The magnetorheological elastomer has a similar cross-linked amorphous structure to SR, but the matrix amount was much lower in relation to the embedded CIP, limiting the elasticity. Despite the fact that MRE has a low elongation due to its less amorphous structure, which represented toughness, and the CIP, which provided the strength characteristic. Furthermore, the crystallinity embedded in the amorphous matrix has produced secondary interactions [29], increasing the strength of the MRE, as shown by the gradient in the linear regression graph of Figure 4, which is much higher than that of the SR. Both SR and MRE samples exhibited their natural behavior before durability, according to the conceptual underpinnings. 

**Figure 5:**
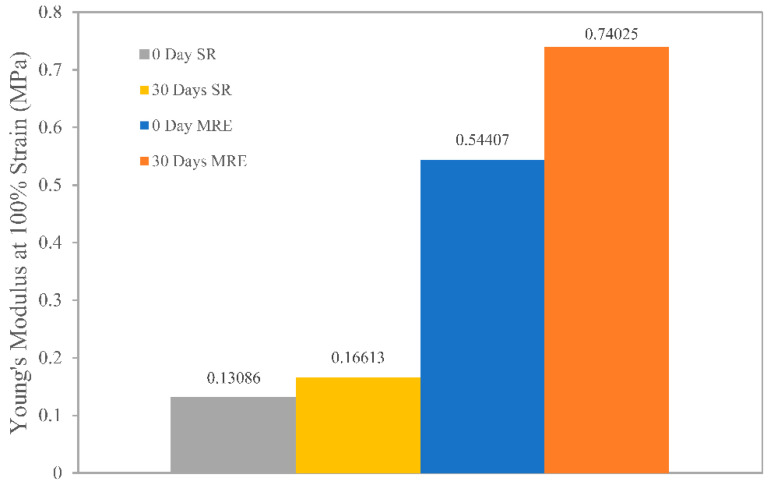
Young’s modulus of SR and MRE under natural weathering conditions.

After 30 days of natural weathering, both SR and MRE samples showed an increase in stiffness with a significant consistency in total strength, indicating the ability to withstand degradation. Inevitably implied that the material was subjected to minimal environmental degradation, most likely as a result of chain scissions and photo-oxidation reactions. Photo-oxidation was attributed to enhanced brittleness and stiffness of the SR and MRE, thus increasing Young’s modulus, as depicted in Figure 5. The Young’s modulus for MRE of 0.74 MPa was measured after 30 days of weathering durability, compared to 0.54 MPa before that condition, indicating that the modulus value increased by at least 35% as a result of natural weathering or environmental stress. Similarly, a 25% increase in Young’s modulus value for SR was observed following the test.

**Figure 6:**
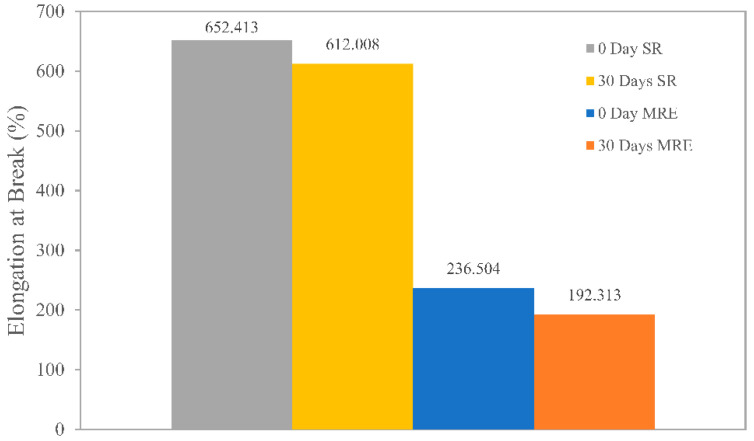
Elongation at break of SR and MRE under natural weathering conditions.

In addition to chain scission, the matrix experienced crystallinity, which could be caused by photodegradation, which rearranged segmented molecules of entangled molecules in the matrix’s amorphous phase. The degradation was more pronounced in MRE and SR due to an increase in surface layer crystallinity, which caused shrinkage when both samples were exposed to UV radiation from the sunlight. As illustrated in Figure 6, those processes reduced the sample’s elongation. Prior to weathering durability, the SR sample had the highest elongation of 650%, which dropped by 10% after that. The ability to elongate was still good even though the SR sample was predominantly composed of an amorphous structure. However, because the amorphous structure in MRE was much lower (≈30%) when combined with environmental stress factors such as photo-oxidation, ozone, crystallization, and UV radiation, MRE exhibited a nearly 20% reduction in elongation. The results obtained were in an agreement with the previous studies conducted using elastomeric polymer [21,22].

### 2.4. The Effects of Natural Weathering on Rheological Properties

**Figure 7:**
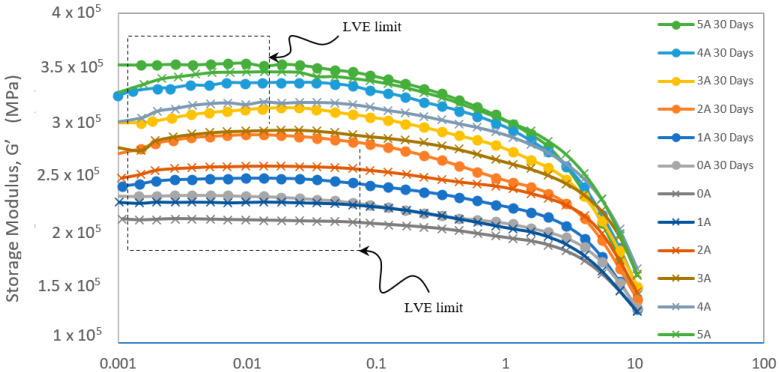
Magnetorheological elastomer storage modulus evaluation before and after 30 days durability under natural weathering conditions.

Based on the tensile test results, the investigation and evaluation of the MRE performance under natural weathering for its rheological behavior was carried out. As previously stated, it was suspected that the degradation of MRE was primarily due to molecular degradation. Because it involved a molecular aspect, rheological analysis was critical to understanding the effect on molecular structure. The storage modulus (G’) representation was then used to evaluate MRE’s ability to store deformation energy. Figure 7 depicts the experimental results of MRE storage modulus behavior under natural weathering durability. The storage modulus behavior was studied at up to 10% of strain, when G’ became strain dependent (within 0.01%) and independent (after 0.1%). The earlier region was called the linear viscoelastic region (LVE) and the characteristic of MRE in this region represented the elasticity and stiffness of the MRE sample. Magnetorheological elastomer G’ has agreed with a similar pattern from the mechanical tensile test under all conditions. The increasing G’ value in the sample indicated that the MRE was becoming stiffer as the magnetic inducement increased. However, the G’ changed only slightly when subjected to high magnetic stimulation. The materials might experience magnetically saturated in high fields. The brittleness of the MRE sample could be seen in the graph as the LVE limit has been reduced by magnetic induce currents ranging from 0 to 5 A. This was consistent with the tensile test results of elongation at break, which decreased after 30 days of weathering exposure due to crystallinity and the shrinkage of the surface from photodegradation.

The storage modulus graph presented in Figure 7 is the first of its kind to be reported on MRE under natural weathering durability, with no artificial or accelerated weathering conditions. Natural weathering in a tropical climate exhibited a distinct degradation process, with dry and wet conditions averaging 27.5 °C and 287.5 mm rainfall with 74.3% humidity, respectively. Previous research [17] on accelerated weathering of MRE found that the G’ decreased by up to 85% with increasing magnetic strength after the accelerated weathering condition. These findings, however, were established under much more aggressive conditions, such as a difference of 120% in temperature and at least a 20% difference in humidity in comparison to the current study. The magnetorheological elastomer matrix might soften and age when exposed to temperatures of up to 60 °C during the accelerated test. Furthermore, continuous exposure to UV radiation and other accelerated parameters may cause MRE to degrade faster and worsen than MRE tested under natural conditions. The obtained result, on the other hand, demonstrated the characteristic of MRE in more intense and harsh conditions outside of the tropical climate. According to another study [25], the differences in the results obtained from natural weathering and accelerated weathering were most likely due to a change in the mechanism leading to oxygen uptake. As a result, the current study on natural weathering conditions provided critical and ideal data for future accelerated weathering studies on MRE, resulting in more reliable and accurate predictions.

## 3. Materials and Methods

### 3.1. Production of MRE

Isotropic MRE based on silicone was used in this study. A closed mold method was used to produce the MRE sample with an elastomeric matrix embedded with 70 wt% magnetizable particles. Soft carbonyl iron particles (CIPs) (d50 = 3.9–5.2 µm, OM grade, supplied by BASF, Germany) were used in this fabrication. At 25 °C, the CIPs were mechanically deposited and blended into a room temperature vulcanization (RTV) silicone rubber (SR) supplied by Nippon Steel Co., Japan. The curing method was carried out in a closed rectangular mold. The mixtures were cured by adding a curing agent (0.1 wt%) and poured inside the mold, then allowed to solidify for 2 h. The curing agent, NS625B (Nippon Steel), acted as a cross-linking agent and determined the desired amorphous MRE matrix characteristic. 

The curing pressure used during the process was evenly distributed, and there was no evidence of gravity segregation. The cured sample has 150 mm length, 150 mm width, and 1 mm thickness of the rectangular sinking section inside the mold under off-state conditions (no external magnetic stimuli). A trimming knife was used to remove excessive matrix bleed-out along the edges of the MRE sheet. Finally, for dynamic oscillatory shear testing, a sheet sample of MRE was punched-cut out using a hollow hole punch tool to the desired diameter of 20 mm and a nominal thickness of 1 mm. A similar technique was used to prepare tensile test samples using tensile cutting dies following ASTM D412, Type C standards. A similar procedure was used to prepare SR sheets using RTV silicone rubber (without CIP), which were used solely to evaluate matrix behavior because they were similar to the matrix used for MRE in this study.

### 3.2. Weathering Exposure Procedure

**Figure 8:**
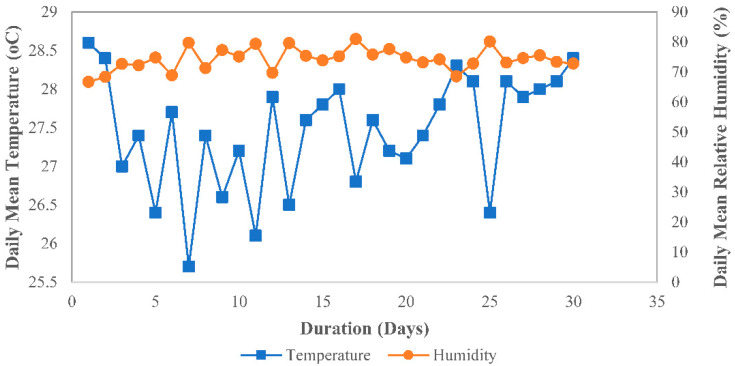
Daily mean temperature and relative humidity in April 2022.

**Figure 9:**
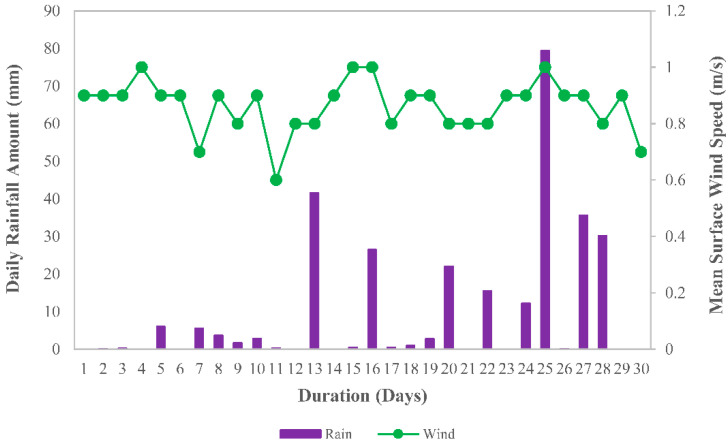
Daily rainfall amount and mean surface wind speed in April 2022.

The natural weathering test was carried out for 30 days from 1st to 30th April 2022 at the Malaysia–Japan International Institute of Technology, Universiti Teknologi Malaysia, Kuala Lumpur, Malaysia. The natural weathering procedure followed the established test standards. The exposed specimens were MRE sheet samples arranged in an expose caged rack in an open area not obstructed by other objects facing south (3.1727, 101.7208)/(3.10′21.77194″ N, 10.43′14.8794″ E). To serve as a reference to the similar matrix used in the MRE, batches of SR sheets (without CIP) were prepared and exposed similarly. Finally, to determine the degree of degradation, the specimens were collected 30 days after being exposed to environmental effects. Before proceeding to further experimental evaluation, the specimens were cleaned with a cleanse cloth and left in the air for 24 h at room temperature. Figure 8 and Figure 9 depict the weather conditions during the sample’s exposure to the tropical climate. The data were obtained from the Malaysian Meteorological Department, Kuala Lumpur, Malaysia.

Throughout the sample exposure, the highest 24 h mean temperature recorded was 28.6 °C on the first day of exposure, and the lowest temperature was 24.6 °C on day 25, which also happened to be the day with the highest daily rainfall amount recorded at 79.4 mm. Daylight in April 2022 was measured with an average ultraviolet index (UV index) of 6 to 7, placing it in the high index category. Furthermore, the humidity and wind speed readings revealed an archetypal tropical climate, which was typically hot, very humid, and wet.

### 3.3. Mechanical Properties by Uniaxial Tensile Test

The samples were tested for quasi-static uniaxial tensile properties before and after natural weathering using a universal testing machine, Shimadzu AGX-S, Kyoto, following ASTM D 412. Dumbbell specimens were cut from the sheets using tensile cutting dies following the same test standard. A constant cross-head speed of 50 mm/min was used and the tests were performed at controlled room temperature (25 °C).

### 3.4. Rheological Analysis via Oscillation Shear Mode

The storage and loss modulus behavior of MRE before and after weathering conditions were measured using an oscillation parallel plate rheometer in oscillatory shear mode (Physica MCR 302, Anton Paar Company, Graz, Austria). A rotary parallel disc plate diligently preloaded the sample in the center of the stationary parallel base plate (pp20 rod). The equipment was configured according to standard operating procedures, and the sample was then subjected to a constant normal force of 5.9 N to circumvent the plane slip surface. The temperature was kept constant at 25 °C throughout the test by a Viscotherm VT2, Anton Paar attached to the rheometer. The obtained storage modulus was then used to calculate the energy of the MRE as a result of elastic deformation. The main reason for measuring the energy stored and dissipated in the MRE was to better understand the degradation due to natural weathering, which affects its lifetime performance.

### 3.5. Fourier-Transform Infrared Spectroscopy

To assess the extent of chemical degradation, weathered MRE and SR sheets were subjected to Fourier-Transform Infrared spectroscopy analysis. Using a sharp cutting knife, the MRE samples to be tested via FTIR were broken down into tiny pieces. The Perkin Elmer Frontier spectrometer, USA, was used for the FTIR testing. The spectrum was obtained at 650–4000 cm^−1^ in transmitted mode with an 8 cm^−1^ resolution.

### 3.6. Morphological Observation

Scanning electron microscopy (SEM) (Tescan Vega 3, Brno–Kohoutovice, Czech Republic) was used to investigate the physical aspects of the samples’ unexposed and exposed areas. Before the experimental evaluation, the primary focus was on the sample surface area before and after exposure to the natural environment. The surface condition was examined for damage, cracks, or significant vicissitudes. The cross section of the initial sample’s state should be closely examined for the distribution of the particles inside the matrix. Samples were prepared for microstructure analysis by cutting the sample into segments with an approximate surface proportion of 1 mm × 10 mm. Following the mechanical properties assessment, similar procedures were applied to the fractured sample. The fractured samples’ surfaces were examined to determine the characteristic of the quasi-static tensile loading used during the test. The sample cross section was located in the gauge length area of both fractured parts of the sample. Image disturbance was reduced constructively using a sputter coating machine to apply a conductive coating process to the specimen. The surface of both samples was sputter-coated with a thin layer of gold at approximately 1 nm thickness using an auto fine sputter coater device (NS800, Novatic Scientific, Singapore).

## 4. Conclusions

Understanding the durability performance of MRE under natural weathering requires complex and critical studies of the weathering process and the mechanism of degradation. Furthermore, the relationship between the process and degradation, and morphological changes is still under scientific debate. The condition of the mechanical and rheological properties of MRE, as well as its interaction with the outdoor environmental surroundings, has been studied and investigated in this study. The morphological observations revealed that surface crystallinity and shrinkage were responsible for the development of the erosion and flaws lines, which increased the modulus by 35%, while decreasing elongation performance by 20%. Magnetorheological elastomer storage modulus from the rheological evaluation was consistent with mechanical test data at an increasing pattern for each magnetic stimulation. Photo-oxidation events of the amorphous cross-linked molecular structure were primarily responsible for the discovery of elementary onset ozone microcracking occurrences in MRE samples. The findings should be noted as useful information for demonstrating the MRE’s durability against natural weathering in tropical climates, as well as a useful reference for future research in determining solutions to develop new MREs with better resistance to environmental weathering conditions. Overall, the findings accommodated in this current work and its microscopic complexion could be useful for future similar research with a longer test perpetuation to better present lifetime performance. More round-robin tests for MRE at different weathering conditions or climates would be beneficial to lay the groundwork for competence and reliability data collection, allowing for the development of related possible test standards. MRE has obvious applications, but from an industrial perspective, there is a general lack of weathering durability information, and it is still unestablished. More information in this field is required to reassure the industry and entice it to invest, to which the current study’s findings have contributed in part.

## Data Availability

The raw/processed data required to reproduce these findings cannot be shared at this time as the data also form part of an ongoing study. In the future, however, the raw data required to reproduce these findings will be available from the corresponding authors.
